# Unlocking the Potential of the Elderly Population in Serbia: A Modeling Study on Musculoskeletal Disorders and Associated Factors

**DOI:** 10.3390/jcm13216541

**Published:** 2024-10-31

**Authors:** Diana Radovic, Milena Santric-Milicevic, Dejan Nikolic, Tamara Filipovic, Jovan Ducic, Ljubica Nikcevic, Milica Jovicic, Ivan Tulic, Goran Tulic

**Affiliations:** 1 Institute of Rehabilitation, 11000 Belgrade, Serbia; diana.radovic.bg@gmail.com (D.R.); tamarabackovic@gmail.com (T.F.); jmilica713@gmail.com (M.J.); 2 Faculty of Medicine, University of Belgrade, 11000 Belgrade, Serbia; milena.santric-milicevic@med.bg.ac.rs (M.S.-M.); jovanducic98@gmail.com (J.D.); ljubicanikcevic@yahoo.com (L.N.); tulic95ivan@gmail.com (I.T.); tulic05@gmail.com (G.T.); 3 Laboratory for Strengthening Capacity and Performance of Health System and Workforce for Health Equity, Institute of Social Medicine, 11000 Belgrade, Serbia; 4 Department of Physical Medicine and Rehabilitation, University Children’s Hospital, 11000 Belgrade, Serbia; 5 Special Hospital for Cerebrovascular Disorders “Sveti Sava”, 11000 Belgrade, Serbia; 6 Institute for Orthopaedic Surgery and Traumatology, University Clinical Center of Serbia, 11000 Belgrade, Serbia

**Keywords:** elderly, musculoskeletal disorders, sociodemographic characteristics, physical functioning, predictors

## Abstract

**Background/Objectives:** A properly functioning musculoskeletal system is imperative for human well-being at every stage of life, including at an older age. This study’s aim was to assess the relationship between sociodemographic and physical functioning variables and the presence of individual musculoskeletal disorders (MSDs), MSD comorbidity, and multimorbidity, as well as to determine factors that are independent predictors of the presence of MSDs in people over 65 years old. **Methods:** This population-based study included 3701 participants aged 65 years and older. Data on individual MSDs addressed cervical and lumbosacral spine regions and degenerative joint disease (arthrosis). The subjects were categorized into four groups: those without any diseases; those with one MSD; those with two MSDs (comorbidities); and those with three MSDs (multimorbidities). The sociodemographic and physical functioning variables were analyzed. **Results:** Females were more likely to have MSDs (two: OR 1.95 and three: OR 2.25) than men. Elderly people aged 75 and above were 1.49 times more likely to have three MSDs. Elderly people with elementary school education were more likely to have MSDs (two: OR 1.34 and three: OR 2.06) than those with high school/university education. The low-income population was 2.47 times more likely to have three MSDs. Individuals with partial activity limitations because of health problems had greater chances of having one, two, or three MSDs (OR 1.60, 1.59, and 1.94, respectively), and elderly individuals with severe limitations had an OR of 1.43, 2.17, and 4.12, respectively. Individuals with some/many difficulties in walking up or down 12 steps were more likely to have MSDs (two: OR 2.26 and three: OR 2.28). **Conclusions:** The significant predictors of experiencing a single MSD, MSD comorbidity, or MSD multimorbidity include residing in the Serbian capital city and having limitations in activities due to health problems. A significant predictor of having a single MSD or MSD comorbidity is residing in the northern region of Serbia. A significant predictor of MSD comorbidity is residing in the southeastern region of Serbia. Significant predictors of MSD comorbidity or MSD multimorbidity include female gender, an elementary school educational level, and experiencing difficulty in walking up or down 12 steps. Significant predictors of MSD multimorbidity are being 75 years of age and above and having a lower income.

## 1. Introduction

A properly functioning musculoskeletal system is imperative for individual well-being at every stage of life, including at an older age. Emphasizing the maintenance of a healthy musculoskeletal system and physical functionality throughout life and into old age is a matter of social priority. This is underscored by the projections of the United Nations Department of Economic and Social Affairs, which indicate that by 2050, the global population of individuals aged 65 years and older will surpass 1.5 billion. This estimate represents a twofold increase from the reported 703 million people in 2019 [1]. Serbia is in the midst of a demographic and epidemiological transition, given that approximately one-fifth of the population is over the age of 65, and there is an observable shift from acute infectious diseases to the presence of multiple chronic conditions among individuals, including musculoskeletal disorders (MSDs), in this age group [2,3]. Projections spanning the next two decades indicate that the proportion of Serbian individuals aged 80 and above is expected to double, reaching 7.44 percent by the year 2040 [3]. In this specific context, it is expected that the prevalence of MSDs will escalate, leading to higher demands and costs for health and care services [4]. Comprehensive estimates encompass the full spectrum of over 150 diverse MSDs, impacting joints, muscles, bones, ligaments, tendons, and the spine [5]. At the global level, high disability and low lethality rates were predicted to be major contributors to MSD-related disability-adjusted life years [6]. It has been stressed that, combined, MSDs account for approximately one-fifth of the total years lived with disability worldwide [7], significantly impairing the health of older adults [8]. Furthermore, MSDs are emerging as an important component of multimorbidity worldwide [9] and are expected to become a long-term concern in the future. This highlights the urgent need to screen factors associated with single MSDs, comorbidity, and multimorbidity in adults to help maintain a healthy musculoskeletal system.

A multidimensional approach to MSDs was found to be pertinent, suggesting a biopsychosocial perspective, which entails the interaction of body structure and function with personal and environmental factors [10]. As individuals grow older, there is a natural decline in both muscle mass and strength [11]. This highlights the importance of monitoring and safeguarding both aspects of physical functioning. Moreover, aging is linked to changes in bone mass, mineral content, bone shape, and geometry, as well as degenerative changes in ligaments, tendons, and joint capsules, which increases the susceptibility to partial or complete disruptions [12]. This may lead to a higher risk of fractures and reduced healing capacity [12]. According to the 2011 census in Serbia, 4.9% of those aged 65 and older need assistance with daily activities, while a higher percentage, 15–18%, require support for instrumental activities [3]. There is a strong relationship between painful musculoskeletal conditions and a reduced capacity to engage in physical activity, which leads to functional decline, frailty, loss of independence, and reduced well-being [13]. A study conducted in Serbia revealed that musculoskeletal diseases (MSDs) place a substantial burden on elderly people primarily due to the presence of associated pain and the costs incurred for pain management [14]. The abovementioned findings underscore the critical need for heightened focus and research investment in this area to address the challenges faced by elderly individuals with MSDs. Although the Serbian Aging Strategy for the 2024–2030 period recognizes the importance of palliative care and violence prevention [15], it fails to address the presence of numerous MSDs among the elderly population and their impact on their mobility and, thus, hinders the promotion of healthy aging and understanding of the aging process. It is essential to first identify specific groups that could benefit from targeted interventions. These interventions could include education and training for self-help, new diagnostic approaches, and drug treatments for managing MSDs. However, increasing evidence suggests that manual therapy can have significant pain-inhibiting effects and hypoalgesia, which, in turn, may play a role in improving function and reducing disability in the elderly population [16]. It is also crucial to specify the factors associated with MSDs occurring independently and those occurring alongside other MSDs (comorbidity) or multiple MSDs (multimorbidity). The sociodemographic profile and physical functioning patterns of the elderly population with MSDs provide valuable insights for policymakers looking to prioritize musculoskeletal health and the capacity for independent living. The information serves as a pivotal foundation for the development of enduring strategies to promote sustainable health and well-being for elderly communities within our society, aligning with the objectives outlined in the United Nations Agenda for Global Development and the World Health Organization Global Strategy and Action Plan on Aging and Health, which was adopted in 2017. Specifically, the strategy focuses on the following five strategic objectives: commitment to action on healthy aging in every country; developing age-friendly environments; aligning health systems to the needs of older populations; developing sustainable and equitable systems for providing long-term care (homes, communities, and institutions); and improving measurement, monitoring, and research on healthy aging [17].

The aim of our study was to assess the relationship between sociodemographic and physical functioning variables and the presence of individual MSDs, MSD comorbidity, and MSD multimorbidity, as well as to determine factors that are independent predictors of the presence of musculoskeletal disorders in people over 65 years old.

## 2. Materials and Methods

### 2.1. Study Design, Participants, and Data Sources

This population-based study included 3701 people aged 65 years and older (27.3% of the representative sample of Serbian residents in 2019). Population data for this secondary analysis were taken from anonymous and electronically based original data sources in the National Study “2019 Survey of the Population Health in Serbia”, which was financed by the Ministry of Health of the Republic of Serbia [18]. The study followed the methodology and instruments of the European Health Interview Survey (EHIS) wave 3 [19]. The study was approved by the Institutional Ethical Board of the Faculty of Medicine, University of Belgrade (No. 25/VII-7, Date: 8 July 2024).

### 2.2. Selection Criteria for Participation in the Study

The study included data on elderly individuals aged 65 years and older who lived in private households in the Republic of Serbia during the data gathering of the national “2019 Survey of Population Health in Serbia” [18]. Data from those who resided in collective households, residents of geriatric institutions, and those who refused to participate in the study were excluded. To inform on regional sociodemographic disparities and facilitate tailored regional health planning, financing, and resource allocation, the main stratum of the investigated population sample was divided into the five geostatic territories with similar population sizes (i.e., in the nomenclature of statistical territorial units, level 2 (NUTS2)). The NUTS divides each country in the European Union into the following three levels: NUTS 1, major socioeconomic regions; NUTS 2, basic regions (for regional policies); and NUTS 3, small regions (for specific diagnoses). The NUTS is used to collect, develop, and harmonize European regional statistics. In the study, the following four out of a total of five NUTS2 regions in Serbia were analyzed: the northern (Vojvodina), capital (Belgrade), central-western (Šumadija and Western Serbia), and south-eastern (Southern and Eastern) regions. It is worth noting that northern Serbia has the densest road network in Serbia, the capital is the most economically developed area, and central-western Serbia has the most agriculturally and industrially developed regional characteristics. At the same time, the southeastern part is a mountain–valley region with rapid depopulation and de-agrarization. The 2019 health survey was not conducted in the southern NUTS2 region of Kosovo and Metohija.

### 2.3. Musculoskeletal Disorders as Variables of Interest in Modeling

Data on the individual MSDs studied addressed the following pathologies: those of the cervical spine region, lumbosacral spine region, and degenerative joint disease (arthrosis). According to the EHIS, in the national “2019 Survey of the Population Health in Serbia”, the following question was asked: “During the past 12 months, have you had any of the following diseases or conditions?”

1. Arthrosis—degenerative joint disease (does not include arthritis (joint inflammation)).
2. Lower back pain disorders or other chronic back problems.
3. Painful disorders in the cervical spine or other chronic problems with the cervical spine.

The offered answers were yes or no. All medical conditions were self-reported by the studied individuals [18,19].

### 2.4. Sociodemographic Characteristics of the Elderly Population as Input Variables in the Regression Model

The sociodemographic characteristics of the study participants included the following eight variables: gender, age, body mass index (BMI), educational level, marital status, household income, household size, and the regions of the Republic of Serbia. The gender variable included both male and female participants. The participants were divided into the following age groups: 65–74 years, 75–84 years, and 85 years and older [20]. According to the classification by the World Health Organization (WHO), participants were categorized into the following four BMI groups: underweight (<18.50), normal weight (18.50–24.99), overweight (≥25.00), and obese (≥30.00) [21]. For the evaluation of the body mass index (BMI), the body height and weight measurements were taken and calculated in kg/m^2^. In our study, respondents’ educational level of the elderly participants was grouped into three categories according to the interview instructions: elementary school (8 years of education), high school (between 9 and 12 years of education), and university (>12 years of education). Marital status was divided into single (never married, divorced, or widowed) and married/living with a partner. The income quintile group was collected globally at the household level and disseminated by quintile of the income distribution: the first, second, third, fourth, and fifth quintile groups. The Serbian households were ranked into five socioeconomic categories (the richest, rich, middle class, poor, and the poorest). In our study, respondents’ income was divided into three categories: lower (poor and poorest), middle (middle class), and upper (rich and the richest). Household size was categorized into three groups based on the number of members: single, two, and three or more members. The study analyzed four regions of the Republic of Serbia: the northern, capital, central-western, and southeastern regions.

### 2.5. Physical Functioning of the Elderly Population as an Input Variable in the Regression Model

We investigated five categories of physical functioning. The first one was a limitation in daily activities. The question was “Are you limited because of a health problem in activities people usually do?”. The proposed answers were related to musculoskeletal health problems (i.e., MSDs) and were offered on a three-level frequency scale, including no limitations, limited but not severe, and severe limitations. The second part was an index scoring the limitations in personal care activities. It included five questions related to executing the following activities without assistance: eating, lying down and getting out of bed, sitting and getting up from a chair, dressing and undressing, using the toilet, and bathing or showering [22]. For each of these questions, the respondent could choose one of the following four answers: no difficulty, some difficulty, a lot of difficulty, cannot at all/unable. The third part was an index scoring household activities. It included seven questions about performing the following activities without assistance: preparing food, making phone calls, going shopping, taking therapy (medications), light housework, occasional heavy housework, and keeping track of finances, accounts, and other administrative tasks [22]. For each of these questions, the respondent could choose one of four answers: no difficulty, some difficulty, a lot of difficulty, cannot at all/unable. The fourth part addressed walking difficulties. The questions asked were the following: “Do you have difficulty walking half a km on level ground without the use of any aid?” and “Do you have difficulty going up or down 12 steps on your own?”. The proposed answers on a four-point response scale were the following: no difficulty, some difficulty, a lot of difficulty, cannot at all/unable. The last physical activity was addressed using two questions: “In a typical week, on how many days do you walk for at least 10 min continuously to get to and from places?” on a four-point answer scale (never, 1–2 days, 3–4 days, 5 days or more) and “How much time do you spend sitting or reclining during a typical day?” on a four-point response scale (0–2 h, 3–5 h, 6–9 h, 10 h or more).

### 2.6. Statistical Analysis

Categorical variables are presented as absolute and relative frequencies. We used Pearson’s chi-squared test to assess the statistically significant variables (*p* < 0.05). Cell frequencies are compared in each row and in each column. For the identification of factors that were independent predictors of the presence of musculoskeletal disorders, we used univariate multinomial logistic regression and multivariate multinomial logistic regression that included variables from the univariate logistic regression with *p* < 0.05. We used a cross-odds ratio (OR) with a 95% confidence interval (CI) to quantify the strength of the association of significant predictors and musculoskeletal disorders. During the multinomial logistic regression analysis, the subjects were categorized into four groups: those without any disease, those with one disease, those with two diseases (comorbidity), and those with three musculoskeletal disorders (multimorbidity). Furthermore, the potential predictors concerning these four groups were analyzed simultaneously using a group of subjects without diseases as the reference category. Subjects with one, two, and three musculoskeletal disorders were coded with 1, 2, and 3, respectively. Predicted probabilities for the tested variables were extracted from the univariate multinomial regression models, and graphs were constructed using these data. All statistical analyses were performed using IBM SPSS statistics for Windows, version 22. 0 (IBM Corp., Armonk, NY, USA) and Microsoft Excel (Microsoft Office 365 Windows 10).

## 3. Results

### 3.1. Sociodemographic Characteristics of the Elderly Population and Their Relation to Musculoskeletal Disorders

In the elderly participant sample, there were more male and female individuals without MSDs than those exhibiting a singular MSD, MSD comorbidities, and multimorbidities (Table 1).

Among individuals with MSDs, females were significantly more prevalent than males. The proportion of females in the MSD comorbidity group was nearly twice as high as that of males (*p* < 0.001), and the proportion was three times greater in the MSD multimorbidity group (*p* < 0.001). The number of participants in the 65–74 age group was nearly double that in the older age group. However, among individuals with MSDs, the distribution differed, with a higher prevalence of individuals aged 75 years and above exhibiting MSD multimorbidities compared with those in the 65–74 age group (*p* < 0.001). Obesity and underweight were less common than normal weight and overweight among participants. Obesity was significantly more common among individuals with three MSDs (*p* = 0.014). In the sample, there were fewer elderly individuals with elementary school education, but they were significantly more common in the group with three musculoskeletal disorders (MSDs) (*p* < 0.001). Those with a high school education were significantly more common in the group with no MSDs (*p* < 0.001), and those with a university-level education were significantly more common in the group with one MSD (*p* = 0.002). In the sample and all study groups, there was a higher number of married respondents or those living with a partner, except in the group with three MSDs (*p* < 0.001). The majority of elderly people had middle incomes. However, elderly individuals with lower incomes were significantly more frequent among individuals with three MSDs (*p* < 0.001), while those with higher incomes were significantly more frequently found to have no MSDs or a single MSD (*p* = 0.044). Single-person households were most common among study individuals with three MSDs (*p* < 0.001), while those with three or more members in the household most commonly had no MSDs (*p* < 0.001). Elderly people from central-western Serbia were significantly the most frequent with no MSDs (*p* < 0.001) (Table 1).

**Figure 1:**
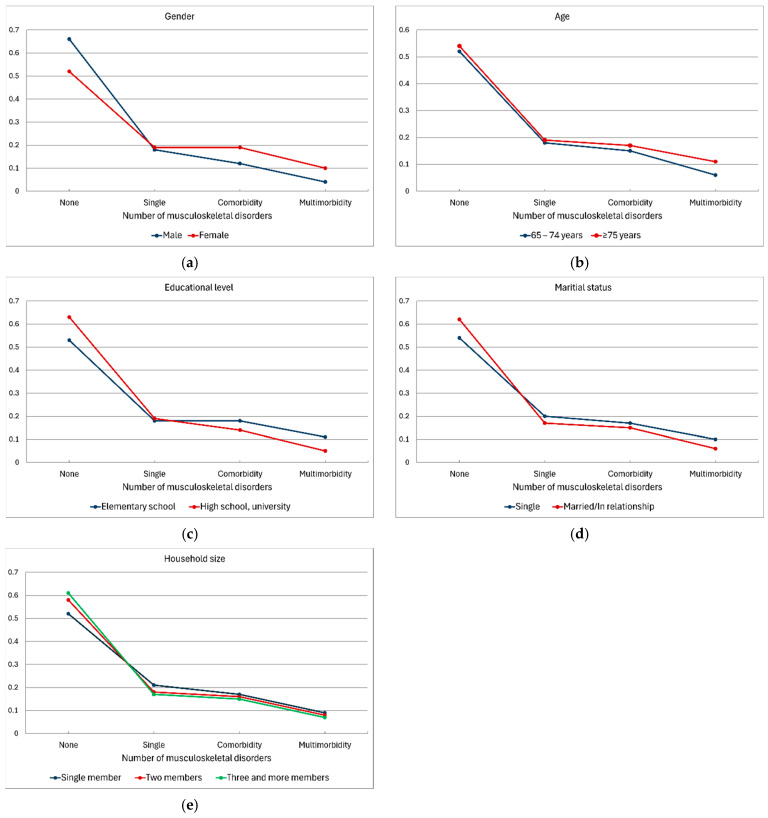
(**a**–**e**) Predicted probabilities for the tested sociodemographic variables in association with the presence of musculoskeletal disorders. (**a**) Gender; (**b**) age; (**c**) educational level; (**d**) marital status; and (**e**) household size.

Figure 1a–e presents predicted probabilities for the tested sociodemographic variables associated with the presence of MSDs.

### 3.2. The Physical Functioning of the Elderly Population and Its Relation to Musculoskeletal Disorders

Elderly people with a single MSD more frequently had no limitations (35.8%) or non-severe limitations (43.3%) in activities they usually performed due to health problems; they had no difficulties in performing personal care activities (72.5%) and household activities (45.0%). Furthermore, they more frequently had no difficulty or some difficulty in walking half a kilometer on level ground without the use of any aids (45.0% and 32.2%, respectively), and they had no difficulty or some difficulty in walking up or down 12 steps (41.3% and 34.4%, respectively). They more frequently performed a 10 min continuous walking activity three or more days weekly (5 or more days (69.3%) and 3–4 days (8.6%)) (Table 2).

Elderly people with MSD comorbidities more frequently had some difficulty performing household activities (34.9%) and sitting or reclining for 0–5 h daily (0–2 h (22.0%) and for 3–5 h (42.7%) (Table 2).

Elderly people with MSD multimorbidities more frequently had severe limitations in performing activities they usually performed due to health problems (45.0%); they had some difficulty, much difficulty, or were unable to perform personal care activities (26.3%, 14.7%, and 5.8%, respectively), and they had much difficulty or were unable to perform household activities (28.1% and 15.7%, respectively), walking half a kilometer on level ground without the use of any aids (29.1% and 23.0%, respectively), and walking up or down 12 steps (34.7% and 22.7%, respectively). Moreover, they walked 10 min continuously on 2 days or less weekly (1–2 days (9.0%) and never (32.0%)), and they sat for 6 and more hours daily (6–9 h (27.5%) and 10 and more hours (18.6%)) (Table 2).

**Figure 2:**
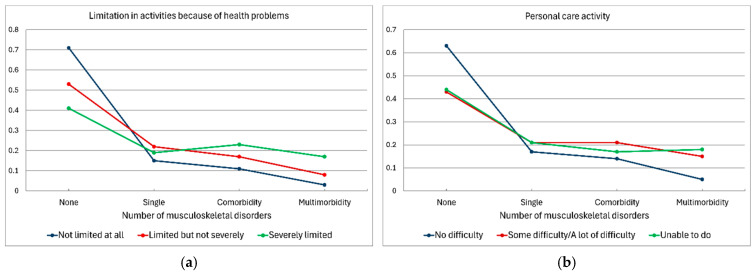

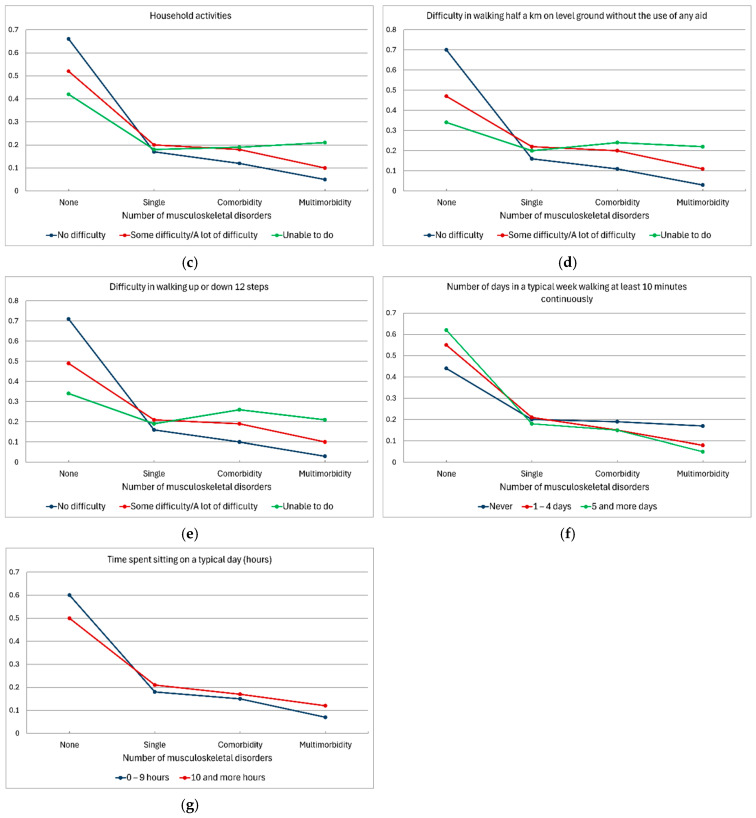
(**a**–**g**) Predicted probabilities for tested physical functioning variables associated with the presence of musculoskeletal disorders. (**a**) Limitations in activities because of health problems; (**b**) personal care activities; (**c**) household activities; (**d**) difficulty in walking half a kilometer on level ground without the use of any aids; (**e**) difficulty in walking up or down 12 steps; (**f**) number of days in a typical week with walking for at least 10 min continuously; (**g**) time spent sitting on a typical day (hours).

Figure 2a–g presents the predicted probabilities for the tested physical functioning variables associated with the presence of MSDs.

### 3.3. Modeling of Musculoskeletal Disorders and Associated Factors

The multinomial logistic regression analysis of sociodemographic variables for the number of evaluated MSDs is presented in Table 3. In the univariate multinomial logistic regression, gender and age were positively associated with one, two, or three MSDs. Women were 1.29, 2, and 3.16 times more likely than men to have one, two, and three MSDs, respectively. The participants were divided into two age groups, 65–74 years and 75 years and older, because the number of respondents in the over-85 group was insufficient for this analysis. Elderly individuals who were aged 75 and above were about 1.3 times more likely to have one or two MSDs and 2.17 times more likely to have three MSDs than those aged 65–74 years. BMI was positively associated with the presence of three MSDs, while income was positively associated with the presence of two and three MSDs. Elderly individuals with elementary school education were 1.5 times more likely to have two and 2.83 times more likely to have three MSDs than those with high school or university education. Those who were single were 1.29 times more likely to have one or two MSDs and 1.91 times more likely to have three MSDs. Households with single members were a significant predictor of the presence of one, two, or three MSDs in comparison with households with three or more members. The elderly population in Serbia’s capital, northern, and southeastern regions was about 1.5 times more likely to have one or two MSDs than the population in the central-western region.

In the multivariate analysis, a significant predictor was gender; women were 1.95 times more likely to have two and 2.25 more likely to have three MSDs than men. Age was also a significant predictor in the multivariate analysis; the elderly persons aged 75 and above were 1.49 times more likely to have three MSDs. Elderly people with elementary school education were 1.34 times more likely to have two and 2.06 times more likely to have three MSDs than those with high school or university education. Lower income was a significant predictor only for the presence of three MSDs. The regions of Serbia were also significant predictors in the multivariate analysis, but not in all categories. In the multivariate analysis, the body mass index, marital status, and household size were insignificant predictors.

In the univariate multinomial logistic regression (Table 4), limitations in activities because of health problems, personal care activity, household activities, difficulties in walking half a kilometer on level ground without the use of any aids, difficulty in walking up or down 12 steps, and the number of days in a typical week in which one walked to get to and from places for at least 10 min continuously were found to be positively associated with the presence of one, two, and three MSDs. In contrast, the time spent sitting in a typical day (in hours) was positively associated with the presence of one and three MSDs.

Limitations in performing everyday activities due to health problems were a significant predictor in all categories, with OR values from 2.03 to 9.72. For the study, answers of some difficulty and much difficulty to questions about personal care activities, household activities, difficulty walking half a kilometer on level ground without using any aids, and difficulty walking up or down 12 steps were united in one group. Personal care activities were significantly associated with some MSDs when we analyzed individuals who did not have difficulties in comparison with those with some difficulties/many difficulties (OR values from 1.81 to 4.10). Household activities were significantly associated with one, two, and three MSDs in all categories (OR from 1.49 to 2.79 in the analysis of no difficulties vs. some difficulties/many difficulties; OR from 1.64 to 5.04 in the analysis of no difficulties vs. inability to do).

Difficulty walking half a kilometer on level ground without the use of any aids was significantly associated with the presence of MSDs; elderly individuals who had some difficulties or many difficulties were 2.09, 2.74, and 5.04 times more likely to have one, two, and three MSDs, respectively. Individuals who were unable to walk half a kilometer on level ground were 2.58, 4.45, and 14.8 times more likely to have one, two, or three MSDs, respectively.

Elderly individuals with one, two, or three MSDs were 1.97, 2.70, or 5.75 times more likely to have some difficulties or many difficulties walking up or down 12 steps, respectively, and they had 2.49, 5.17, or 16.7 times greater chances of being unable to walk up or down 12 steps, respectively (Table 4).

After applying variables that were significantly associated with MSDs in the evaluated models from the univariate into multivariate logistic regression analyses, personal care activities, household activities, difficulty in walking half a kilometer on level ground without the use of any aids, the number of days in a typical week in which one walked to get to and from places for at least 10 min continuously, and time spent sitting on a typical day (hours) were not significant predictors. Limitations in activities because of health problems and difficulties walking up or down 12 steps remained significant predictors in the multivariate analysis.

Elderly people who were partially limited in activities because of health problems had greater chances of having one, two, or three MSDs (OR: 1.60, 1.59, and 1.94, respectively), as did elderly individuals with severe limitations (OR: 1.43, 2.17, and 4.12, respectively). Individuals with some/many difficulties in walking up or down 12 steps were about 2.3 times more likely to have two or three MSDs.

## 4. Discussion

This study assessed the relationship between sociodemographic and physical functioning variables and the presence of a single MSD, two MSDs (comorbidity), and three MSDs (multimorbidity) in individuals aged 65 years and above. This population-based study included 3701 people aged 65 years and older. Data on individual MSDs that were studied addressed the following pathologies: those of the cervical spine region, those of the lumbosacral spine region, and arthrosis—degenerative joint disease (not including arthritis (joint inflammation)). This study used the following sociodemographic characteristics: gender, age, body mass index (BMI), educational level, marital status, household income, household size, and the regions of the Republic of Serbia. The physical functioning variables used in this model were limitations in daily activities, personal care activities, home activities, walking difficulties, and physical activities.

### 4.1. Sociodemographic Predictors of Musculoskeletal Disorders in Elderly People

Considering the sociodemographic predictors, MSDs are more prevalent among females than males. The proportion of females in the MSD comorbidity group was nearly twice as high as that of males and it was three times higher in the MSD multimorbidity group. In our study, we observed that females were 1.29, 1.99, and 3.16 times more likely than males to have one, two, and three MSDs, respectively. Our study showed a higher prevalence of individuals aged 75 and above exhibiting MSD multimorbidities compared with those in the 65–74 age group. In the study of Croft et al., it was noticed that many individuals with knee pain had pain at multiple joint sites [23]. Therefore, it should be assumed that older people, particularly those 75 years and above, will have more MSDs and that this group of the population should be included in regular multidisciplinary and interdisciplinary evaluations in healthcare settings with proper follow-up and optimal treatment interventions. Previous reports also stated that females are more affected by musculoskeletal disorders, such as chronic back pain and chronic arthritis/joint pain [24]. Moreover, a study that was conducted on participants aged 50 years and above showed that chronic musculoskeletal pain in older European adults was more frequent in females [25]. Both neck and lower back pain were reported to be more prevalent in females [26]. Overstreet et al. stated that the role of biopsychosocial factors and mechanical factors, genetics, immunology, and hormones associated with musculoskeletal pain diseases may contribute to gender differences [26]. Our study showed that obesity was significantly more common among individuals with three MSDs. There are two main hypotheses explaining the effect of obesity on painful musculoskeletal disorders [27]. First, it has been hypothesized that joint degradation due to excessive body mass may predispose individuals to develop osteoarthritis, especially in the lower back, knee, and hip joints. Alternatively, pain in MSDs may also predispose individuals to become obese because of reduced activity. Nevertheless, what the present study shows is that obesity is an essential factor when it comes to painful MSDs [27,28]. In our study, elderly people with elementary school education were 1.48 times more likely to have two MSDs and 2.83 times more likely to have three MSDs than those with high school or university education. Studies have shown that the level of education is related to the choice of profession; a low level of education predisposes a subject to be employed in a profession with heavy physical requirements [29]. In a self-reported study of individuals between 50 and 70 years of age, it was observed that an occupation with heavy physical demands was a risk factor for back and neck pain. Self-rated heavy workload was strongly correlated with low levels of formal education [29]. A Danish study on individuals between 60 and 70 years of age pointed out that higher education was associated with fewer painful musculoskeletal disorders [30]. Lower income was significantly more frequent among individuals with three MSDs, while those with higher income significantly more frequently had no MSDs or a single MSD. Lower income was a significant predictor only for the presence of three MSDs. In the study of Hagen et al., it was shown that the most substantial relationship was found between low socioeconomic status and the prevalence of widespread chronic MSDs [31]. Higher income was associated with a lower risk of musculoskeletal pain complaints. The adverse effects on mood and general health stemming from economic stress are well documented. It was proposed that higher income might enable the application of extra care for older individuals with MSDs [28].

Single-person households were most common among study individuals with three MSDs, while those with three or more members in the household most commonly had no MSDs. Older people from central-western Serbia significantly most frequently had no MSDs.

### 4.2. Physical Functioning Predictors of Musculoskeletal Disorders in the Elderly Population

Regarding the physical functioning predictors, our results pointed out that older people were limited in activities to some extent or had severe limitations due to musculoskeletal diseases; half of the respondents had difficulties or could not perform household activities, but about one-third had problems in personal care. Partial (not severe) limitations that were health-related were the most frequent in the group of elderly people with single MSDs, while severe limitations were the most frequent among individuals with MSD multimorbidity. In a univariate multinomial logistic regression, we found that limitations in performing everyday activities due to health problems were a significant predictor in all categories, with OR values from 2.03 to 9.72. Study individuals with multimorbidities had some or many difficulties or could not perform personal care activities. Personal care activities were significantly associated with some MSDs when we analyzed individuals who did not have difficulties vs. those with some difficulty/many difficulties, with OR values from 1.81 to 4.10. Older people with multimorbidities were unable to perform or had many difficulties in performing household activities. Difficulty in walking half a kilometer on level ground without the use of any aids was significantly associated with the presence of MSDs; elderly people who have some or many difficulties were 2.09, 2.74, and 5.04 times more likely to have one, two, and three MSDs. Individuals who could not walk half a kilometer on level ground were 2.58, 4.45, and 14.8 times more likely to have one, two, or three MSDs. Elderly individuals with one, two, or three MSDs were 1.97, 2.70, or 5.75 times more likely to have some or many difficulties in walking up or down 12 steps and had 2.49, 5.17, or 16.7 times greater chances of being unable to walk up or down 12 steps. The number of days per week when elderly people walked to get to and from places for at least 10 min continuously decreased with the increase in the number of MSDs, and elderly people with multimorbidities most frequently performed such activities on 1–2 days per week or never walked. Those with no MSDs performed 3–5 h of daily sitting activities, while elderly people with multimorbidities performed such activities for 6–9 h, and performing such activities for ten or more hours occurred significantly more frequently with multimorbidity. After applying variables that were significantly associated with musculoskeletal disorders (MSDs) in the evaluated models, limitations in activities due to health problems and difficulty in walking up or down 12 steps remained significant predictors in the multivariate analysis. The study of Stamm et al. confirmed that people with musculoskeletal conditions were significantly more often affected by problems with activities of daily living (ADLs) than people without these diseases. The results of these researchers showed that the ADL domain that caused problems for most people was performing heavy household chores, bending or kneeling, climbing up and down stairs without a walking aid, and walking 500 m without a walking aid, which correlated with our results [32]. The InCHIANTI study on elderly people aged 65 years and above pointed out that those with lower back pain (LBP) were more likely to report difficulties in the performance of most ADLs. Older adults affected by LBP may reduce their activity and, over time, progressively lose their functional status and independence [33]. The results of our study substantially confirmed the finding of Di Iorio et al. that the association between LBP and the need for help in mobility to perform ADLs is almost fully attributable to the detrimental effect of MSDs on physical performance. Moreover, another study showed that pain in the knees and hips (osteoarthritis) was significantly associated with lower scores on the Short Physical Performance Battery for assessing lower-extremity physical performance in those aged 65 years and older [34]. Furthermore, it was shown that neck pain in older people is associated with impaired physical performance and fear of falling [35]. Our results agree with those of this study, which showed that pain in the lower extremities is associated with the deterioration of physical function and that damage to the musculoskeletal system affects ADLs [34]. In the MOBILIZE Boston Study population, older people with multisite musculoskeletal pain demonstrated poorer standing balance, which was associated with impaired physical performance and fear of falling [36]. The presence of two or three MSDs in our study could be considered as multiple sites being affected. Previous findings suggested that individuals with multisite pain have poorer lower-extremity mobility performance [36]. The complexity of multisite musculoskeletal pain in older adults is because it is associated with lower-extremity mobility, impairments of the upper extremities, increased risk of falling, and general disability, as well as psychological dysfunction and social factors [37]. Moreover, the positive effects of physical exercise, particularly when supervised, including improved performance, fitness, and vitality in older adults with musculoskeletal impairment were previously described [38]. These findings suggest the importance of regular health and physical fitness checks of older adults by a multidisciplinary team of professionals, timely inclusion in exercise and rehabilitation programs, and interventions to improve overall physical functioning and reduce complications, further declines, and disability. In these times of economic and demographic challenges, it is crucial for policymakers, financiers, civic societies, and non-profit organizations to develop and implement effective support for people living with MSDs. The evidence from this research could help in a more comprehensive understanding of the health problems and needs of the elderly population in Serbia and worldwide and, thus, represent a kind of contribution to the creation of joint actions to promote healthy aging in countries with pronounced demographic changes. In this sense, the identified needs could be used to plan the necessary health services and health workers, primarily in the fields of physical medicine and rehabilitation, physical therapy, and public health.

### 4.3. Study Limitations

The study is subject to several limitations. Firstly, as a cross-sectional study, it lacks the ability to establish causative relationships between variables and the study outcomes. Additionally, while the sample is representative, the generalizability of our findings may be confined to the household population. To gain a more comprehensive understanding of the subject, future research efforts should encompass individuals residing in institutional settings, such as nursing homes and homes for elderly people. Unfortunately, this was not feasible in our study, as it constituted a secondary analysis of national health survey data collected periodically within households by the Ministry of Health. At the same time, the Ministry of Labor, Employment, Veterans, and Social Affairs plays a pivotal role in assessing individuals’ welfare in communal housing or institutional settings, and those datasets were beyond our analysis. The study findings are based on selected social and demographic variables and individual physical functioning that were reported. Therefore, it is imperative to scrutinize medical records to augment the overall comprehension of the variables. Subsequent research should employ a cohort study design to extend the monitoring of health indicators over time and encompass risk factors, health behavior, and the utilization of health and care services. This approach would facilitate the formulation of hypotheses for timely prevention and health intervention. Given that our findings are predicated on EHIS indicators for the physical functioning of the elderly population and reflect the cultural and policy contexts of MSDs in the country, our study may yield valuable insights into MSDs within similar international settings.

## 5. Conclusions

Regarding sociodemographic variables, a significant predictor of single MSDs, MSD comorbidities, or MSD multimorbidities was residing in the Serbian capital city. A significant predictor of single MSDs or MSD comorbidities was residing in the northern region of Serbia, while a significant predictor of MSD comorbidities was residing in the southeastern region of Serbia. Significant predictors of MSD comorbidities or MSD multimorbidities were female gender and an elementary school educational level. Significant predictors of MSD multimorbidities were being 75 years of age and above and having a lower income.

Regarding physical functioning, a significant predictor of single MSDs, MSD comorbidities, or MSD multimorbidities was having limitations in activities due to health problems, while a significant predictor of MSD comorbidities or MSD multimorbidities was having difficulty in walking up or down 12 steps.

## Figures and Tables

**Table 1:** Sociodemographic differences among the elderly population concerning musculoskeletal disorders: no disorders, single disorders, comorbidity, and multimorbidity.

Sociodemographic Variables	Elderly Individuals Grouped by Number of Musculoskeletal Disorders, n (%)					
	None (n = 2166)	Single (n = 681)	Comorbidity (n = 576)	Multimorbidity (n = 278)	Total (n = 3701)	*p*
Gender						
Males	1096 (50.6)	302 (44.3)	196 (34.0)	68 (24.5)	1662 (44.9)	<0.001
Females	1070 (49.4)	379 (55.7)	380 (66.0)	210 (75.5)	2039 (55.1)	<0.001
*p*	0.576	0.003	<0.001	<0.001	<0.001	
Age						
65–74 years	1414 (65.3)	409 (60.1)	344 (59.7)	129 (46.4)	2296 (62.0)	<0.001
75–84 years	601 (27.7)	222 (32.6)	182 (31.6)	118 (42.4)	1123 (30.4)	<0.001
≥85 years	151 (7.0)	50 (7.3)	50 (8.7)	31 (11.2)	282 (7.6)	0.065
*p*	<0.001	<0.001	<0.001	<0.001	<0.001	
BMI						
Underweight	26 (1.4)	10 (1.8)	9 (2.0)	3 (1.4)	48 (1.6)	0.78
Normal weight	593 (32.5)	163 (29.4)	125 (28.1)	57 (26.0)	938 (30.8)	0.078
Overweight	737 (40.2)	224 (40.4)	184 (41.3)	81 (37.0)	1226 (40.2)	0.751
Obesity	470 (25.7)	158 (28.5)	127 (28.5)	78 (35.6)	833 (27.3)	0.014
*p*	<0.001	<0.001	<0.001	<0.001	<0.001	
Educational level						
Elementary school	919 (42.5)	311 (45.7)	301 (52.3)	188 (67.6)	1719 (46.5)	<0.001
High school	927 (42.8)	248 (36.4)	197 (34.2)	66 (23.7)	1438 (38.8)	<0.001
University	318 (14.7)	122 (17.9)	78 (13.5)	24 (8.6)	542 (14.7)	0.002
*p*	<0.001	<0.001	<0.001	<0.001	<0.001	
Marital status						
Single	838 (38.8)	306 (45.0)	259 (45.0)	152 (54.7)	1555 (42.1)	<0.001
Married/living with a partner	1324 (61.2)	374 (55.0)	317 (55.0)	126 (45.3)	2141 (57.9)	<0.001
*p*	<0.001	0.009	0.016	0.035	<0.001	
Income						
Lower	387 (17.9)	149 (21.9)	122 (21.2)	81 (29.1)	739 (20.0)	<0.001
Middle	1406 (64.8)	418 (61.4)	370 (64.2)	166 (59.7)	2360 (63.7)	0.176
Upper	373 (17.2)	114 (16.7)	84 (14.6)	31 (11.2)	602 (16.3)	0.044
*p*	<0.001	<0.001	<0.001	<0.001	<0.001	
Household size						
Single member	335 (15.5)	139 (20.4)	112 (19.4)	61 (21.9)	647 (17.5)	<0.001
Two members	773 (35.7)	242 (35.5)	209 (36.3)	101 (36.3)	1325 (35.8)	<0.001
Three or more members	1058 (48.8)	300 (44.1)	255 (44.3)	115 (41.7)	1729 (46.7)	<0.001
*p*	<0.001	<0.001	<0.001	<0.001	<0.001	
Region						
Northern	484 (22.4)	170 (25.0)	146 (25.3)	58 (20.9)	858 (23.2)	0.220
Capital	476 (22.0)	174 (25.6)	142 (24.7)	61 (21.9)	853 (23.0)	0.184
Central-Western	712 (32.9)	170 (25.0)	141 (24.5)	81 (29.1)	1104 (29.9)	<0.001
Southeastern	494 (22.8)	167 (24.5)	147 (25.5)	78 (28.1)	886 (23.9)	0.169
*p*	<0.001	0.985	0.981	0.117	<0.001	

BMI—body mass index. Data missing range: for BMI (296 or 8.0%); for educational level (2 or 0.05%); and for marital status (5 or 0.1%).

**Table 2:** Physical functioning of the elderly population according to musculoskeletal disorders: no musculoskeletal disorders, single musculoskeletal disorders, comorbidity, and multimorbidity.

Physical Functioning Variables	Elderly Population Grouped by the Number of Musculoskeletal Disorders, n (%)					
	None (n = 2166)	Single (n = 681)	Comorbidity (n = 576)	Multimorbidity (n = 278)	Total (n = 3701)	*p*
Limited due to health problems (musculoskeletal disease) in activities people usually perform						
No limitation	1161 (53.7)	243 (35.8)	179 (31.1)	48 (17.3)	1631 (44.1)	<0.001
Limitation but not severe	692 (32.0)	294 (43.3)	223 (38.7)	105 (37.8)	1314 (35.5)	<0.001
Severe limitation	311 (14.4)	142 (20.9)	174 (30.2)	125 (45.0)	752 (20.3)	<0.001
*p*	<0.001	<0.001	0.056	<0.001	<0.001	
Difficulties in performing personal care activities						
No difficulty	1789 (82.6)	494 (72.5)	395 (68.6)	148 (53.2)	2826 (76.4)	<0.001
Some difficulty	267 (12.3)	138 (20.3)	126 (21.9)	73 (26.3)	604 (16.3)	<0.001
A lot of difficulty	69 (3.2)	30 (4.4)	40 (6.9)	41 (14.7)	180 (4.9)	<0.001
Cannot do at all/Unable to do	40 (1.8)	19 (2.8)	15 (2.6)	16 (5.8)	90 (2.4)	<0.001
*p*	<0.001	<0.001	<0.001	<0.001	<0.001	
Difficulties in performing household activities						
No difficulty	971 (55.2)	250 (45.0)	178 (38.4)	67 (27.7)	1466 (48.5)	<0.001
Some difficulty	523 (29.7)	170 (30.6)	162 (34.9)	69 (28.5)	924 (30.6)	0.157
A lot of difficulty	190 (10.8)	103 (18.6)	89 (19.2)	68 (28.1)	450 (14.9)	<0.001
Cannot do at all/Unable to do	76 (4.3)	32 (5.8)	35 (7.5)	38 (15.7)	181 (6.0)	<0.001
*p*	<0.001	<0.001	<0.001	<0.001	<0.001	
Difficulties in walking half a km on level ground without the use of any aid						
No difficulty	1380 (63.7)	306 (45.0)	215 (37.3)	61 (21.9)	1962 (53.0)	<0.001
Some difficulty	505 (23.3)	219 (32.2)	170 (29.5)	72 (25.9)	966 (26.1)	<0.001
A lot of difficulty	182 (8.4)	99 (14.6)	123 (21.4)	81 (29.1)	485 (13.1)	<0.001
Cannot do at all/Unable to do	98 (4.5)	56 (8.2)	68 (11.8)	64 (23.0)	286 (7.7)	<0.001
*p*	<0.001	<0.001	<0.001	0.325	<0.001	
Difficulties in walking up or down 12 steps						
No difficulty	1274 (58.9)	281 (41.3)	186 (32.5)	47 (17.0)	1788 (48.4)	<0.001
Some difficulty	570 (26.3)	234 (34.4)	171 (29.8)	71 (25.6)	1046 (28.3)	<0.001
A lot of difficulty	218 (10.1)	109 (16.0)	139 (24.3)	96 (34.7)	562 (15.2)	<0.001
Cannot do at all/Unable to do	102 (4.7)	56 (8.2)	77 (13.4)	63 (22.7)	298 (8.1)	<0.001
*p*	<0.001	<0.001	<0.001	<0.001	<0.001	
Days per week spent walking to get to and from places for at least 10 min continuously						
5 or more days	1587 (77.0)	450 (69.3)	370 (68.9)	137 (53.5)	2544 (72.6)	<0.001
3–4 days	171 (8.3)	56 (8.6)	45 (8.4)	14 (5.5)	286 (8.2)	0.432
1–2 days	92 (4.5)	46 (7.1)	28 (5.2)	23 (9.0)	189 (5.4)	0.003
never	211 (10.2)	97 (14.9)	94 (17.5)	82 (32.0)	484 (13.8)	<0.001
*p*	<0.001	<0.001	<0.001	<0.001	<0.001	
Hours spent sitting or reclining on a typical day						
0–2 h	417 (21.2)	123 (20.0)	114 (22.0)	48 (19.4)	702 (21.0)	0.784
3–5 h	952 (48.4)	253 (41.2)	221 (42.7)	85 (34.4)	1511 (45.1)	<0.001
6–9 h	405 (20.6)	156 (25.4)	119 (23.0)	68 (27.5)	748 (22.3)	0.013
10 and more hours	194 (9.9)	82 (13.4)	64 (12.4)	46 (18.6)	386 (11.5)	<0.001
*p*	<0.001	<0.001	<0.001	<0.001	<0.001	

Data missing range: for limitations in activities because of health problems (5 or 0.1%); for difficulties in performing personal care activities (1 or 0.03%); for difficulties in performing household activities (80 or 2,2%); for difficulties in walking half a kilometer on level ground without the use of any aids (2 or 0.05%); for difficulties in walking up or down 12 steps (7 or 0.2%); for days per week spent walking to get to and from places for at least 10 min continuously (78 or 2.1%); for hours spent sitting on a typical day (54 or 1.5%).

**Table 3:** Multinomial logistic regression analysis of sociodemographic variables for the number of evaluated MSDs.

	Number of Musculoskeletal Disorder Groups in the Elderly Population		
Predictors	Single Musculoskeletal Disease	Comorbidity—Two Musculoskeletal Diseases	Multimorbidity—Three Musculoskeletal Disease
Univariate multinominal logistic regression, OR (95% CI)			
Gender			
Male	1	1	1
Female	1.29 ** (1.08–1.53)	1.99 *** (1.64–2.41)	3.16 *** (2.38–4.21)
Age			
65–74 years	1	1	1
≥75 years	1.25 * (1.05–1.49)	1.27 * (1.05–1.53)	2.17 *** (1.69–2.79)
BMI			
Normal weight	1	1	1
Overweight	1.11 (0.88–1.39)	1.18 (0.92–1.52)	1.14 (0.80–1.63)
Obese	1.22 (0.95–1.57)	1.28 (0.97–1.69)	1.73 ** (1.20–2.48)
Underweight	1.40 (0.66–2.96)	1.64 (0.75–3.59)	1.20 (0.35–4.09)
Educational level			
High school, university	1	1	1
Elementary school	1.14 (0.96–1.36)	1.48 *** (1.23–1.78)	2.83 *** (2.17–3.69)
Marital status			
Married/living with a partner	1	1	1
Single	1.29 ** (1.09–1.54)	1.29 ** (1.07–1.55)	1.91 *** (1.48–2.45)
Income			
Upper	1	1	1
Middle	0.97 (0.77–1.23)	1.17 (0.90–1.52)	1.42 (0.95–2.12)
Lower	1.26 (0.95–1.67)	1.40 * (1.02–1.91)	2.52 *** (1.63–3.90)
Household size			
Three or more members	1	1	1
Single member	1.46 ** (1.16–1.85)	1.39 * (1.08–1.79)	1.66 ** (1.19–2.32)
Three or more members	1	1	1
Two members	1.10 (0.91–1.34)	1.12 (0.91–1.38)	1.19 (0.90–1.58)
Region			
Central-Western	1	1	1
Capital	1.53 ** (1.20–1.95)	1.51 ** (1.16–1.95)	1.13 (0.79–1.60)
Northern	1.47 ** (1.15–1.87)	1.52 ** (1.18–1.97)	1.05 (0.74–1.50)
Southeastern	1.42 ** (1.11–1.80)	1.50 ** (1.16–1.95)	1.39 (1.00–1.93)
Multivariate multinomial logistic regression, OR (95% CI)			
Gender			
Male		1	1
Female		1.95 *** (1.49– 2.55)	2.25 *** (1,52–3.32)
Age			
65–74 years			1
≥75 years			1.49 *(1.05–2.10)
Educational level			
High school, university		1	1
Elementary school		1.34 * (1.03–1.75)	2.06 *** (1.40–3.04)
Income			
Upper			1
Middle			1.43 (0.78–2.61)
Lower			2.47 ** (1.28–4.75)
Region			
Central-Western	1	1	1
Capital	1.62 ** (1.18–2.22)	1.87 ** (1.31–2.66)	1.71 * (1.06–2.77)
Northern	1.48 * (1.09–2.01)	1.91 *** (1.37–2.66)	1.17 (0.74–1.86)
Southeastern	1.34 (0.99–1.81)	1.74 ** (1.26–2.40)	1.35 (0.89–2.05)

BMI—body mass index; * *p* < 0.05; ** *p* < 0.01; *** *p* < 0.001.

**Table 4:** Models of musculoskeletal disorders and associated physical functioning factors for elderly people when grouped by the number of present musculoskeletal diseases (single, comorbidity, and multimorbidity).

	Elderly People Grouped by the Number of Musculoskeletal Disorders		
Predictors	Single Musculoskeletal Disease	Comorbidity—Two Musculoskeletal Diseases	Multimorbidity—Three Musculoskeletal Diseases
Univariate multinomial logistic regression, OR (95% CI)			
Limitations in activities due to health problems (musculoskeletal disease)			
Not limited at all	1	1	1
Limited but not severely	2.03 *** (1.67–2.46)	2.09 *** (1.68–2.60)	3.67 *** (2.58–5.23)
Severely limited	2.18 *** (1.71–2.78)	3.63 *** (2.85–4.63)	9.72 *** (6.81–13.9)
Personal care activities			
No difficulty	1	1	1
Some difficulty and A lot of difficulty	1.81 *** (1.45–2.24)	2.24 *** (1.80–2.78)	4.10 *** (3.13–5.38)
No difficulty	1	1	1
Cannot do at all/Unable to do	1.72 (0.99–3.00)	1.70 (0.93–3.11)	4.84 *** (3.64–8.84)
Household activities			
No difficulty	1	1	1
Some difficulty and A lot of difficulty	1.49 *** (1.22–1.81)	1.92 *** (1.55–2.38)	2.79 *** (2.05–3.79)
No difficulty	1	1	1
Cannot do at all/Unable to do	1.64 * (1.06–2.53)	2.51 *** (1.63–3.86)	5.04 *** (3.69–6.87)
Difficulty in walking half a km on level ground without the use of any aids			
No difficulty	1	1	1
Some difficulty and A lot of difficulty	2.09 *** (1.74–2.50)	2.74 *** (2.25–3.34)	5.04 *** (3.69–6.87)
No difficulty	1	1	1
Cannot do at all/Unable to do	2.58 *** (1.81–3.66)	4.45 *** (3.17–6.26)	14.8 *** (9.84–22.2)
Difficulty in walking up or down 12 steps			
No difficulty	1	1	1
Some difficulty and A lot of difficulty	1.97 *** (1.65–2.37)	2.70 *** (2.20–3.30)	5.75 *** (4.11–8.04)
No difficulty	1	1	1
Cannot do at all/Unable to do	2.49 *** (1.75–3.54)	5.17 *** (3.70–7.22)	16.7 *** (10.9–25.7)
Number of days in a typical week walking to get to and from places for at least 10 min continuously			
Five or more days	1	1	1
1–4 days	1.37 * (1.06–1.76)	1.19 (0.90–1.58)	1.63 * (1.11–2.40)
Five or more days	1	1	1
I never carry out such physical activities	1.62 *** (1.25–2.11)	1.91 *** (1.46–2.50)	4.50 *** (3.31–6.13)
Time spent sitting on a typical day (hours)			
0–9 h	1	1	1
10 or more hours	1.41 * (1.07–1.86)	1.29 (0.95–1.74)	2.09 *** (1.47–2.98)
Multivariate multinomial logistic regression, OR (95% CI)			
Limitations in activities because of health problems			
Not limited at all	1	1	1
Limited but not severely	1.60 *** (1.24–2.07)	1.59 ** (1.19–2.12)	1.94 ** (1.23–3.05)
Severely limited	1.43 * (0.93–2.20)	2.17 *** (1.41–3.32)	4.12 *** (2.34–7.25)
Difficulty in walking up or down 12 steps			
No difficulty		1	1
Some difficulty and A lot of difficulty		2.26 *** (1.51–3.37)	2.28 ** (1.22–4.27)

* *p* < 0.05; ** *p* < 0.01; *** *p* < 0.001.

## Data Availability

Data supporting obtained results can be obtained from the first author upon reasonable request.
